# Predicting Protein–Protein Interaction Sites Using Sequence Descriptors and Site Propensity of Neighboring Amino Acids

**DOI:** 10.3390/ijms17111788

**Published:** 2016-10-26

**Authors:** Tzu-Hao Kuo, Kuo-Bin Li

**Affiliations:** 1 Institute of Biomedical Informatics, National Yang-Ming University, Taipei 112, Taiwan; kzvito@gmail.com; 2 Office of Information Management, National Yang-Ming University Hospital, Yilan 260, Taiwan

**Keywords:** Protein–Protein Interaction, intrinsically-disorder protein, machine learning algorithms

## Abstract

Information about the interface sites of Protein–Protein Interactions (PPIs) is useful for many biological research works. However, despite the advancement of experimental techniques, the identification of PPI sites still remains as a challenging task. Using a statistical learning technique, we proposed a computational tool for predicting PPI interaction sites. As an alternative to similar approaches requiring structural information, the proposed method takes all of the input from protein sequences. In addition to typical sequence features, our method takes into consideration that interaction sites are not randomly distributed over the protein sequence. We characterized this positional preference using protein complexes with known structures, proposed a numerical index to estimate the propensity and then incorporated the index into a learning system. The resulting predictor, without using structural information, yields an area under the ROC curve (AUC) of 0.675, recall of 0.597, precision of 0.311 and accuracy of 0.583 on a ten-fold cross-validation experiment. This performance is comparable to the previous approach in which structural information was used. Upon introducing the B-factor data to our predictor, we demonstrated that the AUC can be further improved to 0.750. The tool is accessible at http://bsaltools.ym.edu.tw/predppis.

## 1. Introduction

The study of Protein–Protein Interactions (PPIs) has a crucial role in biology, medicine and the pharmaceutical industry. PPIs can be investigated from two aspects: The interaction partners of a specific protein and the amino acid residues participating in a given PPI. Information about a protein’s interaction partners allows scientists to construct protein interaction networks, such as signaling pathways, which in turn facilitate the understanding of many biological and clinical observations [1,2]. Identifying protein interaction sites and thereby uncovering the microscopic mechanism of how two proteins interact, on the other hand, is especially relevant in the drug development industry [3].

Despite the advancement in experimental techniques, massive and systematic studies on PPI interfaces remain a challenging task. Direct exploration of the interaction interface requires advanced and often costly techniques, such as X-ray crystallography, where the sophisticated experimental setup and limitations in biological conditions pose new challenges for researchers in the field. Computational approaches, although unable to provide direct evidence, as many of the experimental counterparts do, have become increasingly important due to the availability of the massive amount of data that have been accumulated lately [4].

Based on the lifetime of a protein complex, PPIs can be categorized into two types [5,6,7], the permanent and the transient interactions. A permanent interaction is usually stable, suggesting that the involved molecules of the complex rarely separate. Transient interactions, in contrast, allow proteins to form a dynamic association and, therefore, are more commonly seen in mediating biological functions.

In protein interaction networks, the transient protein interactions have a role in maintaining functions of hub proteins, the proteins locating on the center of a subnetwork and, therefore, holding a large number of interaction partners [7,8,9]. Recent studies further suggest that eukaryotic hub proteins have a common feature called intrinsic disorder, a dynamic nature of conformation allowing a protein to interact with many partners [10,11]. Transient interactions were also reported to be associated with linear motifs. Linear motifs are short sequence fragments, often two to eight residues in length, that are known to regulate PPIs, and are mostly found in structurally-unstable loop regions due to the flexibility of the motifs. Although transient PPIs have more functional roles in a biological system, their conformational property hinders their experimental investigation, because unstable proteins are difficult to crystallize and, thus, studied by X-ray crystallography.

Existing methods for predicting PPI sites can be summarized into two categories: The template-based and the intrinsic-based approaches [12]. The template-based approaches involve a comparison of proteins with and without structural data. Being dependent on available templates, those methods perform less satisfactorily when the protein in question presents significant divergence from the proteins with known structures. Without comparing the unknown protein to the extant templates, an intrinsic-based approach extracts features from experimental data and then performs prediction using the features.

The intrinsic-based approaches can be further categorized into sequence-based and structure-based ones. They differ in the features taken into consideration for the prediction. The sequence-based PPI-site prediction only considers features that are available by analyzing proteins’ amino acid sequences. Many sequence-based predictors take advantage of the idea that PPI sites are more conserved than the rest of the protein surface [13,14,15,16,17]. Other methods [18,19] incorporate predicted structural information (e.g., surface accessibility and secondary structure). A recent review [12] suggested that, other than the two types of information mentioned above, sequence-based methods must investigate alternative sources of information to further improve the prediction accuracy.

Reports also indicate that sequence-based methods often have challenges in predicting PPIs if neither of the partner proteins has sequence-wise similar proteins deposited in the training dataset [20,21,22]. Although this type of pair-input predictions is not entirely identical to ours, i.e., predicting PPI sites using a single protein sequence, it clearly shows that homology or similarity-based approaches have intrinsic limitations.

The structure-based PPI-site prediction, albeit more accurate in prediction performance, is only applicable to cases in which the relevant protein structures are available. In reality, due to the intrinsic disorder property, structures of protein complexes involving transient PPIs are more difficult to find.

Two common strategies for protein functional analyses were incorporated into our study: The amino acid physicochemical properties and the sequence conservation. The AAindex [23,24,25,26] is a public database describing more than five hundred amino acid property indices, including hydrophobicity, polarity, bulkiness, etc. It has been reported that protein interaction domains can be identified by analyzing the distribution of hydrophobic amino acid residues [27]. The amino acid property indices can also be indirectly implemented into a discrete model called pseudo amino acid composition [28,29,30,31,32,33], where the amino acid occurrence frequency, as well as the difference in physicochemical properties between neighboring residues are both considered. Evolutionary features extracted from homologous proteins have been applied to predicting PPI sites [34,35,36]. Given a group of homologous proteins, the variation within equivalent residue positions can be observed by aligning those sequences. Evolutionarily-conserved sequence regions are often considered as the functionally-important sites and, hence, serve as candidates in predicting PPI site.

CAPRI (Critical Assessment of PRedictedInteractions) is an experiment testing the ability of docking algorithms to predict the mode of association of two proteins based on their three-dimensional structures [37]. The CAPRI management group provides a benchmark dataset as a form of Protein–Protein complexes, known as the targets. Although predicting PPI sites from protein sequences does not require three-dimensional structures, the CAPRI targets still serve a role as an independent testing dataset.

Given all of the considerations, in this work, we first investigated the characteristics of transient PPIs, then proposed a computational tool to predict PPI sites using the discovered sequence features. We adopted the intrinsic-based approach, in the hope that by not requiring 3D protein structures, our sequence-based method may have a wider applicability.

To facilitate the usefulness of this work, we follow a common guideline for designing sequence-based statistical predictors [38]. This guideline provides a basic framework for many bioinformatics software tools, for example, see the recent publications [31,39,40,41,42,43,44,45,46,47,48,49]. It contains the following recommendations: (1) a benchmark training and testing dataset must be carefully constructed; (2) a mathematical representation of protein sequences must be introduced in such a way that the intrinsic biological properties can be effectively translated into numerical features; (3) a robust machine learning system should be employed; (4) an objective cross-validation experiment must be adopted to evaluate the predictor [21,22]; and (5) the predictor should be deployed as a publicly accessible web site.

## 2. Results

As can be seen in Table 1, the learning and predicting process faces an unbalanced training problem, with the number of non-interacting sites about three-times as many as the number of the interacting counterparts. Given such a dataset, a learning algorithm would have been encouraged to label everything with the majority class. To address the unbalanced learning at the data level, we adopted an under-sampling approach [50] where all of the interacting sites, and a randomly-selected subset of non-interacting sites were used in the training process. A major drawback of the under-sampling scheme is the potential loss of information contained in the ignored samples. This loss of information is not expected to be serious in our case, because the majority class, that is the class of non-interacting sites, underwent a cleaning process in which noisy samples were excluded by the Relative Accessible Surface Area (RASA) filtering process described in the Materials and Methods.

To reduce the chance that proteins in the training and in the testing sets might share high sequence similarity, thereby resulting in unreliable cross-validation [21,22], a 30% identity threshold was used to remove redundant protein chains.

Ten-fold cross-validations on the training set were used to test the performance of the classifier. This is a technical compromise to reduce the training load on computer time. However, it should be noted that among the approaches examining the accuracy of a statistical learning model, the jackknife test is considered the most rigorous and objective one that always yields a unique result for a given benchmark dataset [38]. In a ten-fold cross-validation, the training dataset is randomly partitioned into ten equally-sized subsamples. The validation process consists of ten iterations. In each iteration, a single subsample is retained as the validation data, while the remaining nine subsamples are used as the training data. Each of the ten subsamples is used exactly once as the validation data.

The performance of the training models were evaluated by the Area Under the receiver operating characteristic Curve (AUC). A Receiver Operating Characteristic (ROC) curve is a plot of the true positive rate as a function of the false positive rate. AUC can be interpreted as the expected proportion of the positive samples ranked, by the predictor, before uniformly-drawn random negatives. An AUC of 0.5 is generally considered the baseline performance of a random predictor. Likewise, an AUC of 1.0 indicates that, given a positive and a negative sample, the predictor always gives a higher score for the positive one, suggesting that the predictor is perfect [51].

**Figure 1:**
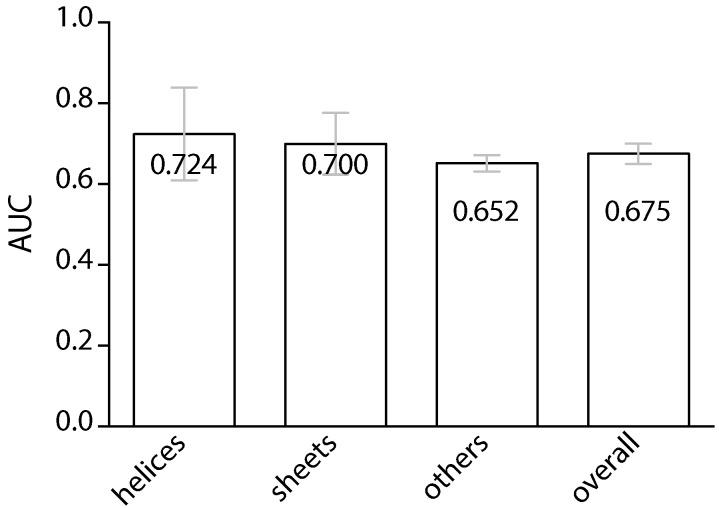
Performance (as measured by AUC) of the proposed protein interaction site predictor, using ten-fold cross-validation. The left three box plots represent the AUC values for amino acid sites located on the three respective secondary structural regions. The right-most box plot represents the overall performance.

The result of the prediction performance, as seen in Figure 1, indicates that the average AUC of the ten-fold cross-validation is 0.675. Taking into consideration that both the first and the second-stage classifications were performed by one of the three classifiers, each corresponding to the predicted secondary structure of the target amino acid site, the AUC for individual types of amino acid sites is also listed. The AUCs for sites predicted to locate on helices, sheets and others are 0.724, 0.700 and 0.652, respectively. It is worth noting that, in the training samples, amino acid sites that are predicted to locate on neither the helical nor the sheet regions account for approximately 60%, according to Table 1. As can be seen in Figure 1, the performances for helical and extended sites are clearly higher than those of other sites, indicating the potential significance of the secondary structure-dependent sequence features, such as the preference of neighboring interacting sites.

Comparing with a structure-based prediction tool [52] that had been evaluated by the same set of protein heterocomplexes, our method relies on sequence features exclusively and, therefore, does not benefit from the availability of the B factors, the temperature factors, that can only be retrieved from the PDB data. The comparison result is tabulated in Table 2. Note that using 12 Å between two C*α* atoms as the threshold, Liu has defined approximately 4600 PPI sites out of the 130 protein chains. On the other hand, using 5 Å between any two atoms as the threshold, we found 3786 PPI sites over the same dataset. The 30% discrepancy between the two figures might be held accountable for the difference in the precision, as it measures the percentage of true predictions among all positive predictions. The lower performance regarding the precision indicates that more false positive predictions could have been made due to our method being purposely designed not to exploit structural information.

To demonstrate the performance of our method on CAPRI targets, we took six protein chains that were selected by a previous method named LORIS [53]. Their PDB IDs are 1S70_A, 1S70_B, 3FM8_A, 3FM8_C, 3Q87_A and 3Q87_C. The maximum sequence identities between a CAPRI target and any of the 128 training chains is 32.1%, averaging over the six targets, indicating in most cases that the CAPRI targets are sequence dissimilar to the training proteins.

Altogether 1324 amino acid residues were included in the evaluation. Applying our definition of PPI sites to those residues resulted in a total of 260 interacting residues. PredPPIS (Predictor for Protein–Protein Interaction Sites) achieved an averaged MCC (Matthews Correlation Coefficient) and F-measure of 0.109 and 0.278, respectively. For comparison purpose, LORIS has a reported overall MCC of 0.115 and F-measure of 0.299. However, it should be noted that, due to the inconsistent definition of PPI interface adopted by PredPPIS and LORIS, respectively, direct performance comparison would not be very easy.

Since user-friendly and publicly-accessible web-servers represent the future direction [54] for developing practically more useful predictors and will significantly enhance their impacts [55,56], we have provided a web-server for the new method presented in this paper, as done in many recent works [39,43,48,49,57,58,59,60]. The server, named PredPPIS, provides a graphical user interface for users to submit their sequences and receive the predictive results. Upon submission of a protein sequence in FASTA format into the text box area, a job code will be generated for users to retrieve the results at a later time, allowing asynchronous job execution. The web-server is available at http://bsaltools.ym.edu.tw/predppis.

## 3. Discussion

This article describes a tool, named PredPPIS, for the prediction of Protein–Protein interaction sites using only sequence information. Although PredPPIS does not require a PDB (Protein Data Bank [61]) data file as its input and, thus, is not dependent on the structural characteristics of protein complexes, it is worth investigating the implication of introducing structural properties, such as the B-factors, on the performance of the prediction.

The B-factor or the temperature factor is considered as an indication of the fluctuation of an atom in a protein. Atoms with low B-factors belong to the well-ordered part of the structure, whereas a large B-factor suggests high mobility of an atom. For interacting proteins, the interface residues are less flexible than the rest of the protein surface and, therefore, are often associated with small B-factors [62]. Given that the B-factor for each atom of a protein is available as part of the PDB file format, the inclusion of B-factors as a prediction feature has been shown to improve the accuracy for predicting PPI sites [52].

**Figure 2:**
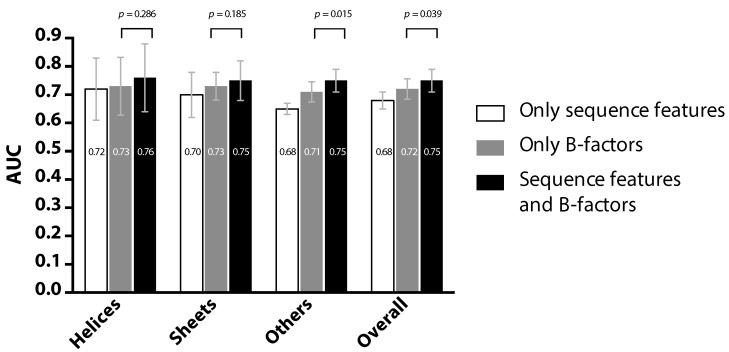
Comparison of the prediction performance using three inputs: sequence features alone, the structural information (the B-factors) alone and both.

**Figure 3:**
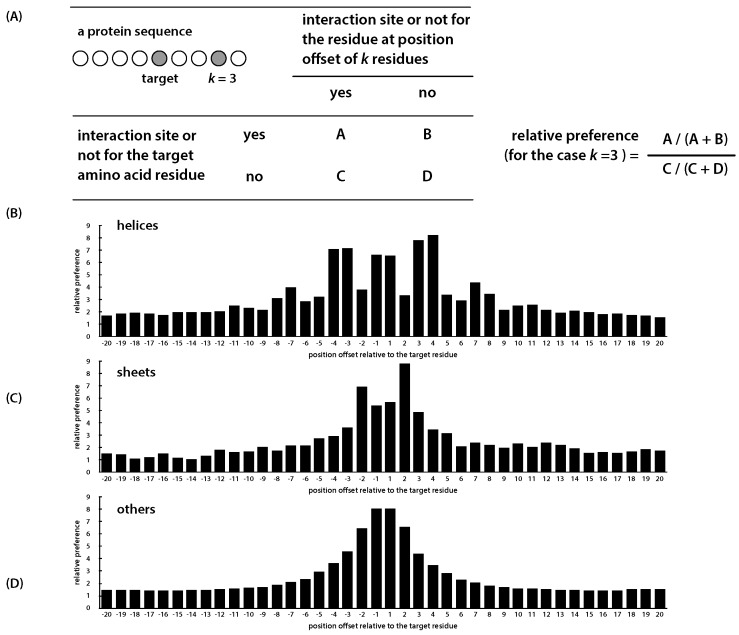
The relative preference of interaction sites among neighboring residues. (**A**) The definition of the relative preference: amino acid residues are represented by circles; the gray filled circles indicate the target amino acid and the neighboring one that is three residues away from the target (i.e., *k* = 3); (**B**–**D**) the relative preferences for interaction sites located on the three secondary structural regions, computed using the training protein sequences.

Figure 2 compares the prediction performance (represented in AUC) of three experiments, each employing the same classification and cross-validation scheme, but involving different feature sets. The first experiment was performed by using the proposed PredPPIS, where the previously-described 36 sequence features were the only input. The second experiment excluded all sequence features and only considered the B-factors of the C*α* atoms, available from PDB data files. The third experiment included the 36 sequence features, as well as the B-factors. By cross examining the results of Experiments 1 and 2, it is clear that PredPPIS was able to achieve comparable performance in the helical regions (0.72 vs. 0.73, in AUC), but not in other regions. This is possibly due to the neighboring effect being more obvious in the helical regions, according to our study (see Figure 3) and, hence, contributing to the prediction performance. Considering all three experiments, although incorporating B-factors into PredPPIS indeed improved the prediction performance, in cases where protein structures, and thus the B-factors, are not available, our method still extracts useful information out of the sequence features.

Multiple-stage predictors are not uncommon in biological sequence analysis; for example, a two-stage classifier where a support vector machine and a Bayesian classifier were used to identify the Protein–Protein interface residues [63]. In that work, the SVM classifier performed the initial prediction by encoding amino acid residues using 20-bit vectors (one bit for each letter representing one of the 20 amino acids). The succeeding Bayesian classifier gave the final prediction by exploiting the observation that interface residues tend to form clusters in a protein. Our classifier also involves two classification stages. The first classifier took the input of the various sequence features and produced scores indicating the initial estimate of the probability of amino acid positions being interaction sites. The sequence features consist of the conservation scores, the amino acid’s physicochemical properties, the relative accessible surface area and the information concerning whether a site is predicted to be located on a disordered region. It is the second stage, where the effect of neighboring positions comes into play, that differentiates our method from the previous ones.

The rationale for the second-stage classifier lies in that the neighboring effect in our work is modeled by a new measurement named the relative preference, inspired by the concept of relative risk in epidemiology. Let residue *i* be the current predicting target and residue i+k be the neighboring amino acid that is *k* residues away from the predicting target. The relative preference models how much more likely that residue *i* would be considered as an interaction site, if the neighboring residue at position i+k is also an interaction site. In other words, the neighboring effect is considered to be dependent on the distance between two residues in the protein sequence. In general, the further apart they are, the weaker the effect can be expected. Nonetheless, as shown in Figure 3, in helical regions, residues at certain distances have a stronger neighboring effect than those at other distances. In brief, one must be able to estimate the probability of the residue i+k being an interaction site, the exact purpose of the first-stage classifier, in order to improve the prediction of the target residue at position *i*, which is to be achieved by the second-stage classifier. In practice, the probability of the residue at position i+k, output by the first-stage classifier, can be considered as a factor adjusting the significance of the neighboring effect contributed by residue i+k toward residue *i*.

**Figure 4:**
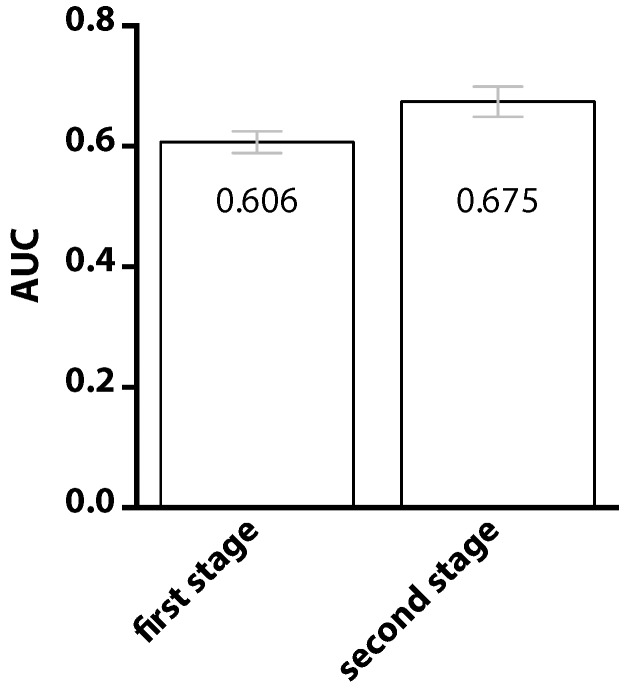
Comparison of the prediction performance using only the first-stage classifier and both the first- and the second-stage classifiers. The neighboring effect, that is the prediction of PPI site can be improved if we knew whether a site’s neighbors are PPI sites or not, is incorporated in the second-stage classifier.

To highlight the importance of the two-stage classification, comparisons are drawn before and after performing the second-stage classification. Results are presented in Figure 4. After introducing the second-stage classifier, the value of AUC was increased from 0.606 to 0.675, revealing that information about the positional preference of interacting sites does indeed benefit the prediction. The improvement is attributable to the observation that neighboring residues are not equally important. Neighbors at the positions with greater relative preferences are expected to be more crucial, at least for the purpose of classification. Given a target residue, if one of its neighbor, located at a position with high relative preference, happens to receive a high predicted probability of being an interaction site, the target itself will be considered to be involved in PPI with higher confidence.

**Figure 5:**
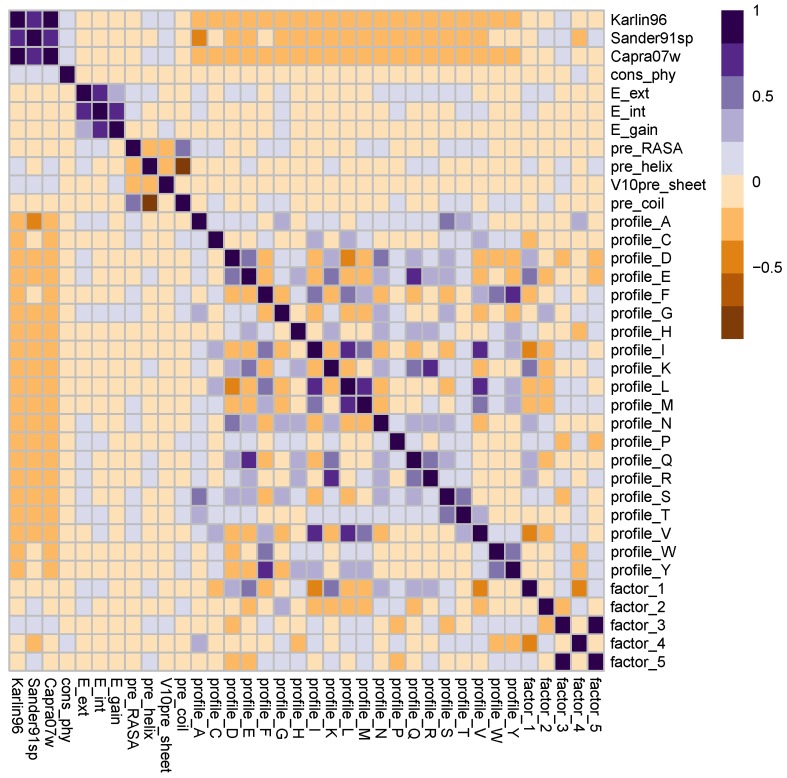
Overview of the correlation coefficients between all of the pairs of the sequence features used in the study.

Disregarding the features related to the neighboring effect, each amino acid residue is represented by 36 sequence features, divided into five categories: (1) orthogonal amino acid indices, five features; (2) Position-Specific Scoring Matrix (PSSM) profiles, 20 features; (3) predicted secondary structures, four features; (4) tendency of being located on disordered regions, three features; and (5) sequence conservation, four features. To investigate the interrelationships among them and, thus, give insight into how many of them are redundant, we computed the pairwise correlations for all features. The result is summarized in Figure 5.

The five amino acid indices, except factor 3 and factor 5, are orthogonal to one another. Moreover, no significant correlation was observed between them and other sequence features. The 20 PSSM features, each representing one type of amino acid, demonstrates considerable dependencies. For example, the PSSM scores for tyrosines, phenylalanines and tryptophans are strongly correlated, due to the higher chance of substitutions among the three amino acids. To keep all possible variability, we decided to keep all 20 scores in the feature set.

Three of the four conservation scores are positively correlated with one another, with the mutational behavior score [64] being the obvious exception. The correlation between the four conservation scores and the PSSM scores is weak, justifying the decision to keep both of them. The same observation goes to the three and the four features originated from predicting the intrinsic disorder and secondary structures, respectively.

Our prediction strategy is not without limitations. First, finding reliable datasets of multimeric proteins is a challenging task. Not only the hetero-complexes are underrepresented in PDB, they are also redundant and often biased towards antibody-antigen or enzyme-inhibitor complexes. Furthermore, problems concerning crystallization conditions or multiple structures solved by NMR spectroscopy highlight the uncertainty in identifying biologically-relevant protein complexes [65]. The list of the hetero-complexes used in this study was taken from [52], in which a BLASTCLUST [66] filtering was performed to remove redundant chains. The sampling bias, however, remains to be a challenge for this bioinformatics study.

The lengths of the proteins also exert a potential impact on the prediction performance. Some sequence features are not meant to characterize short peptides. For example, the pairwise energy scores gained by forming intra- and inter-molecular interactions [67] were computed using a parameter of twenty amino acids as the definition of neighboring residues. As a result, our method is not expected to work satisfactorily for proteins with a length shorter than 20 amino acids. Furthermore, although the inclusion of only transient Protein–Protein interaction pairs as the training data may facilitate the prediction performance, determining the interaction types requires a priori understanding of target protein’s kinetic behavior, which itself may involve time-consuming experiments.

## 4. Materials and Methods

### 4.1. Dataset

The experimental data used in this study were taken from a published work [52], which in turn was derived from a previous study [68]. The proteins whose PDB (Protein Data Bank [61]) entries are obsolete were excluded, resulting in a dataset containing 128 protein chains. Their PDB IDs are listed in Table 3. The sequences and structural information were downloaded from PDB. The latter was used to define known PPI sites. Residues whose three-dimensional coordinates are not available or are designated as the modified residues, such as the selenomethionine, were not considered in the study.

Standalone PSI-BLAST [66,70] searches were performed to generate the list of homologous proteins, which in turn are used in preparing sequence features. Because we are to extract information from conserved amino acid sites, we do not distinguish orthologs and paralogs at this stage. Orthologous proteins retain the functionality during the course of evolution, whereas paralogous proteins may acquire new functions [71]. The distinction between the two is important when inferring a protein’s interacting partners [72,73].

Redundant homologs were removed using CD-HIT [74], with an identity threshold specified as 0.70. The remaining homologous sequences were first aligned using ClustalW [75], then subjected to PHYLIP [76] for computing the phylogenetic distances.

The Protein–Protein interaction sites, that is the amino acid residues physically involved in an interaction, lack a clear-cut definition. Common definitions fall into two categories: on the interatomic distances between residues or on the differences in solvent Accessible Surface Area (ASA) when the monomers of a protein complex are separated. An equally important consideration is the threshold chosen for contacts or for the relative change in accessible surface area. The commonly-accepted distance threshold is 12 Å between C*α* atoms or 6 Å between any two backbone or side chain atoms. For the relative change in ASA between the isolated chains and the complex, 4% is a reasonable threshold [65]. In spite of the difference in definition, the same literature reported that the interface residues determined by either of the two strategies overlapped in 97% of the cases.

In this study, the interatomic distance approach was adopted. Specifically, an amino acid residue is to be considered as a PPI interface site provided at least one of its atoms is within 5 Å or less from any other atom of another amino acid residue. This definition is in accordance with previous reports [77,78] and has been used by other studies, as well [12].

### 4.2. Sequence Descriptors

Evolutionary conservation is an important characteristic in identifying PPI sites using sequence features. Although evolutionary conservation is normally represented using Position-Specific Scoring Matrices (PSSMs), generated by PSI-BLAST, other quantitative measurements of residue conservation have also been proposed and were shown to be effective in many areas [79,80].

The first conservation scoring scheme used in this study is the Karlin96 conservation index [81], a statistic determining the degree of conservation at each position in a multiple alignment. Given a multiple alignment of *N* sequences, let *k* denote an aligned residue position and $A_{ik}$ denote the amino acid residue of sequence *i* at *k*. $M(A_{ik}$, $A_{jk}$) denotes the similarity between amino acid $A_{ik}$ and amino acid $A_{jk}$, measured by the BLOSUM-62substitution matrix. $M_{K}$ represents the normalized similarity. The Karlin96 Conservation Index (CI) specifies the normalized similarity averaged over all amino acid pairs in a given multiple alignment.

$$\begin{array}{cc} \mathrm{CI} & =\frac{2}{N(N-1)}\sum_{i}^{N}\sum_{j>i}^{N}M_{K}\left(A_{ik},A_{jk}\right)\\ & M_{K}\left(A_{ik},A_{jk}\right)=\frac{M(A_{ik},A_{jk})}{\sqrt{M\left(A_{ik},A_{ik}\right)M\left(A_{jk},A_{jk}\right)}}, \end{array}$$

M(i,j) is the BLOSUM-62 score between i and j.

The second scoring represents the relative entropy divergence and is referred to as the Capra07wDivergence Score (DS) [82]. The idea is that a position in a multiple alignment that is found to have an amino acid distribution very different from a background distribution, estimated from a large sequence database, is considered to be functionally important. Here, $p_{k}$ denotes the probability estimated from site *k*; *p* is the probability estimated from the multiple alignment; *q* is the probability estimated from a database. $R(p_{k}$, r) is the relative entropy comparing the two probability distributions.

$$\begin{array}{cc} \mathrm{DS} & =\frac{\left(R\left(p_{k},r\right)+R\left(q,r\right)\right)}{2},\\ & r=\frac{\left(p_{k}+q\right)}{2}. \end{array}$$

The third scoring scheme, the Sander91sp, introduces a weighting factor for each pair of sequences in the multiple alignment [83]. Here, $w_{ij}=d(A_{i}$, $A_{j})$ is the distance between two protein sequences in percent.

$$\begin{array}{c} \mathrm{Sander}91\mathrm{sp}=\frac{\sum_{i}^{N}\sum_{j>i}^{N}w_{ij}M_{K}\left(A_{ik},A_{jk}\right)}{\sum_{i,j}w_{ij}}. \end{array}$$

The last scoring scheme is derived from the concept of mutational behavior [79], in which the authors claimed that methods searching functionally-important amino acid sites can be improved by first dividing protein families into subfamilies, then identifying positions that have functional significance only for the whole family. Those positions were named the tree-determinant residues, because they are the main contributing factors determining the phylogenetic relationships. For each sequence position, a Spearman correlation coefficient is computed and considered as the score. A high value of the score indicates that the variation pattern of that position resembles that of the whole protein family, thus suggesting that the position is a tree-determinant position.

An alignment profile, also known as a PSSM, is a 20×N matrix composed of scores representing the conservation patterns for a given multiple sequence alignment of length *N*. The higher the score in an alignment profile, the more conserved the corresponding position is. The alignment profiles in this study were computed using PSI-BLAST [66,70]. The log-likelihood scores in PSSMs were scaled to the [0,1] range using the method described in a previous study [84]. Twenty feature values were generated for each residue position.

For proteins without available three-dimensional structures, information about secondary structures (helix, extended or other) and solvent accessibility has long been exploited in predicting protein interaction sites [65]. In this study, that information was computed by using RaptorX [85] and NetSurfP [86], respectively, resulting in a total of four features for any given residue.

An amino acid index is a vector of 20 numerical values representing a specific physicochemical or biochemical property of the individual amino acids. To apply rigorous statistical analyses to protein sequence data, a common practice is to convert the sequence’s alphabetic letter codes into numerical values using an amino acid index that is considered relevant to the analyses. However, given the 544 amino acid indices collected by the AAindex database, the task of selecting appropriate indices has become a great challenge for researchers. Selecting too few indices can obscure part of the variability in physico- and bio-chemical properties. Too many indices, though, make the result hard to interpret and sometimes risk losing the generality of computational predictions.

To overcome this sequence metric problem, a small set of numerical indices has been proposed where factor analysis was used to summarize the large and interpretable components of amino acid variation [87]. In this study, the five new amino acid indices, referred to as the factor solution scores by Atchley et al., were employed for the numerical representation of protein sequences. The five indices can be found in [87].

Intrinsically-Disordered Proteins (IDPs) are proteins lacking a persistent structure and, thus, have the nature of being dynamic in conformation [88]. It is estimated that up to 35% of eukaryotic proteins contain stretches of greater than 30 residues of intrinsic disorder [89]. With conformational flexibility, IDPs are suggested to be involved in intra- and inter-protein interactions, in which the disorder-to-order, and thus, loss of entropy, transitions upon binding facilitate the specific, but transient Protein–Protein interactions. To incorporate the intrinsic disorder information into our sequence features, a probability score, the ANCHOR score [67], was included. The ANCHOR score estimates the likelihood of a residue being involved in a disordered binding region. The idea is that residues in disordered binding regions lack strong intra-chain interactions; thus, they cannot fold on their own. However, upon interacting with other globular proteins, residues at the disordered binding regions gain enough stabilizing energy and, therefore, are able to fold into a three-dimensional conformation. Specifically, ANCHOR gives three scores for each residue: the tendency of being disordered, the pairwise energy gained by forming intra-molecular interaction and the pairwise energy gained by interacting with a globular protein. All three scores were included in our sequence feature set.

Prior to subjecting candidate amino acid sites to the two-stage prediction, a pre-screening process was performed to ensure that only amino acids on the surface are to be considered. This was achieved by estimating each residue’s RASA using NetSurfP. Sites with a predicted value of RASA smaller than 0.16, the widely accepted threshold [90], were excluded from the candidate list.

Neighboring residues have long been reported to provide valuable information in predicting the amino acid residues in the PPI interface [63,91]. Ezkurdia et al. [65] also noted that taking into consideration the neighboring residues is crucial for characterizing interacting residues. Despite all of these efforts, there has been no systematic study investigating the distribution of interaction sites within a range of neighboring residues.

By analyzing 333 proteins, Ofran and Rost, 2003, reported that 98% of Protein–Protein interface residues have at least one interface residue appeared within four positions. To investigate such a neighboring effect in a more rigorous and quantitative manner, we have performed the following analysis. Given a residue at the sequence position *i*, for the neighboring residue at position i+k, we defined a new index, which is a function of *k* and is named as the relative preference for residues at a distance of *k* residues away from the position *i*, measuring the ratio of the probability of residue at i+k being an interaction site if *i* itself is an interaction site, to the probability of residue at i+k being an interacting site, if *i* itself is not an interaction site. This is similar to the concept of relative risk used in epidemiology, where the risk between two exposures is compared. A large value of this ratio suggests that, if the neighboring residue at a distance of *k* is an interaction site, the residue at the position *i* is more likely to be considered as an interaction than a non-interaction site. In other words, the relative preference estimates how likely two interaction sites that are *k* residues apart occur simultaneously. The definition and the results are shown in Figure 3.

In our analysis, the value of *k* was selected to range from −20 to +20, where a negative value corresponds to a distance in the sequence toward the protein’s N-terminus and a positive value toward the C-terminus. As depicted in Figure 3, the neighboring effects of interaction residues vary with respect to protein’s secondary structures. In helical regions, an interaction site tends to have another interaction site as its neighbor at an interval of three or four and, again, seven or eight residues. In sheet regions, however, an amino acid residue may have more difficulty in cooperating with its neighboring residues due to conformational constraints and, therefore, lack the preference in neighboring.

**Figure 6:**
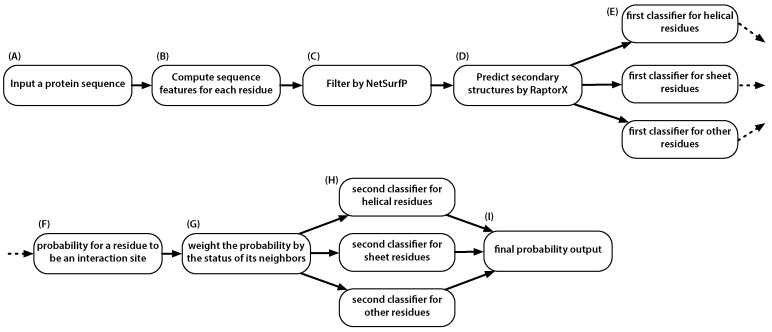
Overview of the steps needed for predicting an amino acid residue site. (**A**) Input a query protein sequence: note that the classifier is to predict PPI sites and, thus, no need to supply two proteins; (**B**) Compute the 36 sequence feature for each residue in the query protein. See the text for details; (**C**) Exclude amino acid sites whose predicted relative accessible surface area are smaller than 0.16; (**D**) Predict the three secondary structures within the query protein; (**E**) Subject the amino acid sites to one of the three classifiers according to the site’s predicted secondary structure. This is necessary because the neighboring effect is dependent on secondary structures; (**F**) Receive the initial probability for each amino acid residue being a PPI site; (**G**) Incorporate the neighboring effect into the initial probabilities by weighting them using data from Table 4; (**H**) Subject sites to one of the three classifiers; (**I**) Obtain the final prediction.

In short, given a candidate amino acid residue, the prediction performance of it being a PPI site could be improved if we knew whether its neighbors are PPI sites or not. This is the major novelty of this work. To implement this idea, we proposed a two-stage predictor, where the first-stage predictor gives predictions to the candidate’s neighbors and the second-stage predictor produces the final prediction. The predictor, named PredPPIS, considers sequence features, including conservation scores, alignment profiles and amino acid indices, in the first stage and the secondary structure-dependent neighboring effect in the second stage. The architecture of the predictor is illustrated in Figure 6.

In the first stage, an extant software tool, RaptorX, was used to estimate the secondary structural components for each amino acid residue. With that information at hand, the site was then subjected to one of the three corresponding sub-classifiers for the first-stage prediction. Note that the first-stage predictor is not an ensemble classifier. Each residue site will be processed by only one of the three sub-classifiers. The statistics of the data are shown in Table 1.

Each candidate residue site is represented by 36 sequence features: (1) 20 features representing the alignment profile; (2) four representing the conservation scores; (3) four representing the predicted relative accessible surface area and the three secondary structures; (4) five representing the amino acid property indices; and (5) three representing the preference of being an interaction site in disordered proteins. To scale the heterogeneous features, the *z*-score normalization was applied to each feature. To incorporate the information provided by neighboring residues, the input feature vector was defined using a sliding window of nine residues. The central residue is the one whose interaction status is to be predicted. The length of the sliding windows was taken from an earlier report [92], in which the optimal length was determined to be nine by analyzing the entropy differences for various window lengths. In summary, each residue is represented by a feature vector consisting of 324 (36×9) features.

Support Vector Machine (SVM), proposed by Vapnik [93], is a statistical learning tool and has been successfully applied to a great number of bioinformatics problems, for example the prediction of Protein–Protein interaction [52,94], DNA methylation sites [95] and the classification of protein subcellular localizations [96]. In this study, LIBSVM [97] was employed to implement the SVM classifying model, using the radial basis kernel and the default grid parameter selection tool.

Once the secondary structure of each amino acid residue was predicted by RaptorX, the feature vector associated with that residue site is then subjected to its corresponding LIBSVM sub-classifier. The output of the first-stage sub-classifiers, provided that LIBSVM’s probability output option was enabled, could be considered as an estimated probability that the input residue site belongs to the positive class, that is the class of interacting amino acid residues.

The sliding windows were set to be nine residues in length, suggesting that the neighboring effect is restricted to the upstream and downstream four residues away from the prediction target. As a result, the integer parameter *k* varies between −4 and +4, excluding zero. The neighboring effect is then modeled by the numerical product between two values, the probability that the residue at position i+k is an interaction site and the relative preference for any residues at the distance of *k*. The data of the relative preferences is listed in Table 4. Given that there are a total of eight possible values of *k*, each residue position now has eight additional features representing the neighboring effects. More specifically, in the second-stage classification, other than the original 324 sequence features specified for a given sliding window consisting of nine residues, an additional 72 features were introduced (eight features for each of the nine residues).

## 5. Conclusions

In this article, we describe a novel computational method for predicting protein interaction sites. The result may provide insight into areas, such as mutant design and the investigation of protein interaction networks. In view of the difficulties of obtaining 3D structures for protein complexes, we adopted a sequence-based approach, in which features for the learning process are exclusively derived from protein sequences. In addition to classical sequence features, such as amino acid conservation and physicochemical properties, we considered the positional preference of interacting sites within a neighboring region in the protein sequence and converted it into sequence features. Experimental results show that our sequence-based method obtains comparable performance over earlier attempts that require structural information. We also demonstrated that incorporating B-factor data into our pipeline may further improve the prediction performance. It is anticipated that the findings derived from this investigation will provide useful clues for further in-depth studies in the problem of predicting Protein–Protein interaction sites.

## Figures and Tables

**Table 1:** Number of amino acid residues in the dataset (128 protein complexes).

Secondary Structure	Interaction Sites	Non-interaction Sites	Total
helix ${}^{a}$	875 (728) ${}^{b}$	5717 (2973)	6592 (3701)
sheet ${}^{a}$	803 (630)	3845 (1619)	4648 (2249)
other ${}^{a}$	2751 (2428)	10,933 (7315)	13,684 (9743)
total	4429 (3786)	20,495 (11,907)	24,924 (15,693)

${}^{a}$ The secondary structures were determined from PDB annotations; ${}^{b}$ the number of amino acid residues with a predicted RASA greater than 0.16 are in parentheses.

**Table 2:** Performance comparison.

Prediction Methods	Recall	Precision	Accuracy	Description
Liu et al. 2010 [52]	0.597	0.407	0.630	B-factors were used as the feature for prediction
this study	0.597	0.311	0.583	only sequence-derived features were used

**Table 3:** The PDB IDs of the protein heterocomplexes.

PDB IDs and the Percentage of Amino Acid Residues in Disorder Regions				
1A4Y_A (6.1) ${}^{a}$	1ABR_A (3.2)	1ABR_B (3.2)	1ACB_I (n.a.) ${}^{b}$	1AHW_B (21.9)
1AHW_C (44.3)	1AK4_A (8.6)	1AK4_D (4.2)	1ATN_D (7.7)	1AVG_H (11.2)
1AVG_I (10.0)	1AVW_B (3.0)	1AVZ_B (25.2)	1AVZ_C (10.8)	1AY7_A (7.3)
1AY7_B (n.a.)	1AZS_B (18.5)	1AZS_C (6.4)	1AZZ_C (3.5)	1B6C_A (4.7)
1B6C_B (n.a.)	1B7Y_A (1.1)	1B7Y_B (6.6)	1BDJ_A (3.7)	1BDJ_B (n.a)
1BGX_T (5.7)	1BI7_A (23.1)	1BI7_B (n.a)	1BI8_B (17.5)	1BMQ_A (9.4)
1BMQ_B (n.a.)	1BP3_A (30.4)	1BP3_B (n.a.)	1BRB_I (n.a.)	1BRS_A (11.5)
1BVK_A (n.a.)	1BVK_C (n.a.)	1BVN_P (8.6)	1BVN_T (n.a.)	1CA0_B (30.0)
1CGI_I (11.4)	1CXZ_B (18.6)	1D4V_A (41.4)	1D4V_B (24.5)	1DAN_L (6.4)
1DAN_T (15.3)	1DAN_U (n.a.)	1DFJ_E (2.1)	1DHK_B (4.9)	1E96_A (3.13)
1E96_B (25.5)	1E9H_B (n.a.)	1EAY_C (10.0)	1EFU_A (2.8)	1EFU_B (4.3)
1ETH_A (3.1)	1ETH_B (5.3)	1FAP_B (8.0)	1FLE_I (31.6)	1FLT_V (18.1)
1FLT_Y (6.4)	1FQ1_A (17.0)	1FSS_B (3.6)	1GFW_A (18.0)	1GFW_B (n.a.)
1GLA_F (3.2)	1GLA_G (5.3)	1GOT_B (9.7)	1GOT_G (2.9)	1GPQ_A (9.5)
1GUA_B (26.5)	1HE8_A (8.4)	1HJA_C (3.2)	1HLU_A (6.4)	1HLU_P (7.2)
1HWH_B (26.3)	1IRA_X (n.a.)	1IRA_Y (3.4)	1ITB_A (2.6)	1JSU_C (31.0)
1KIG_I (16.9)	1KKL_A (2.5)	1KKL_H (6.9)	1KXV_C (0.6)	1L0Y_A (13.9)
1L0Y_B (2.4)	1MAH_A (21.3)	1NOC_A (7.9)	1NOC_B (4.6)	1PDK_A (5.4)
1PDK_B (12.4)	1PYT_A (2.9)	1PYT_B (6.3)	1QBK_B (9.3)	1QBK_C (6.3)
1SGP_E (n.a.)	1SMP_A (6.7)	1SMP_I (3.4)	1SPB_P (n.a.)	1STF_E (3.2)
1STF_I (40.8)	1TAB_I (1.2)	1TCO_A (19.4)	1TMQ_B (6.5)	1UDI_E (14.3)
1UDI_I (n.a.)	1UEA_A (n.a.)	1UEA_B (n.a.)	1UGH_E (n.a.)	1UUZ_A (19.0)
1VAD_A (5.9)	1VAD_B (14.9)	1WQ1_G (9.5)	1XDT_R (9.3)	1XDT_T (33.1)
1ZBD_A (44.3)	1ZBD_B (20.9)	2KAI_B (4.1)	2PCC_A (18.4)	2PCC_B (13.3)
2SIC_I (15.4)	2SNI_I (34.5)	2TEC_E (8.6)	3SGB_I (10.0)	3TGI_I (13.0)
4SGB_I (8.5)	7CEI_A (28.7)	7CEI_B(54.0)		

${}^{a}$ The numbers in parentheses represent the percentage of amino acid residues in disorder regions, estimated by MobiDB [69]; ${}^{b}$ not available.

**Table 4:** The relative preferences for residues located on various positions with respect to the predicting target.

Secondary Structure	−4	−3	−2	−1	+1	+2	+3	+4
helix ${}^{a}$	7.081	7.172	3.817	6.642	6.575	3.359	7.823	8.241
sheet ${}^{a}$	2.924	3.621	6.902	5.391	5.673	8.793	4.859	3.463
other ${}^{a}$	3.620	4.576	6.491	8.035	8.071	6.602	4.428	3.478

${}^{a}$ The secondary structures were predicted by RaptorX.

## Abbreviations

PPI	Protein–Protein Interaction
RASA	Relative Accessible Surface Area
PDB	Protein Data Bank
SVM	Support Vector Machine

## References

[1] Wang X., Wei X., Thijssen B., Das J., Lipkin S.M., Yu H. (2012). Three-dimensional reconstruction of protein networks provides insight into human genetic disease. Nat. Biotechnol..

[2] Chagoyen M., Pazos F. (2016). Characterization of clinical signs in the human interactome. Bioinformatics.

[3] Sudha G., Nussinov R., Srinivasan N. (2014). An overview of recent advances in structural bioinformatics of Protein–Protein interactions and a guide to their principles. Progr. Biophys. Mol. Biol..

[4] Mosca R., Pons T., Céol A., Valencia A., Aloy P. (2013). Towards a detailed atlas of Protein–Protein interactions. Curr. Opin. Struct. Biol..

[5] Acuner Ozbabacan S.E., Engin H.B., Gursoy A., Keskin O. (2011). Transient Protein–Protein interactions. Protein Eng. Des. Sel. PEDS.

[6] Nooren I., Thornton J.M. (2003). Structural characterisation and functional significance of transient Protein–Protein interactions. J. Mol. Biol..

[7] Perkins J.R., Diboun I., Dessailly B.H., Lees J.G., Orengo C. (2010). Transient Protein–Protein interactions: Structural, functional, and network properties. Structure (Lond. Engl. 1993).

[8] Higurashi M., Ishida T., Kinoshita K. (2008). Identification of transient hub proteins and the possible structural basis for their multiple interactions. Protein Sci. Publ. Protein Soc..

[9] Kim P.M., Lu L.J., Xia Y., Gerstein M.B. (2006). Relating three-dimensional structures to protein networks provides evolutionary insights. Science.

[10] Haynes C., Oldfield C.J., Ji F., Klitgord N., Cusick M.E., Radivojac P., Uversky V.N., Vidal M., Iakoucheva L.M. (2006). Intrinsic disorder is a common feature of hub proteins from four eukaryotic interactomes. PLoS Comput. Biol..

[11] Singh G.P., Ganapathi M., Dash D. (2007). Role of intrinsic disorder in transient interactions of hub proteins. Proteins.

[12] Esmaielbeiki R., Krawczyk K., Knapp B., Nebel J.C., Deane C.M. (2016). Progress and challenges in predicting protein interfaces. Brief. Bioinform..

[13] Chen X.W., Jeong J.C. (2009). Sequence-based prediction of protein interaction sites with an integrative method. Bioinformatics.

[14] Wang B., Chen P., Huang D.S., Li J.J., Lok T.M., Lyu M.R. (2006). Predicting protein interaction sites from residue spatial sequence profile and evolution rate. FEBS Lett..

[15] Res I., Mihalek I., Lichtarge O. (2005). An evolution based classifier for prediction of protein interfaces without using protein structures. Bioinformatics.

[16] Lovell S.C., Robertson D.L. (2010). An integrated view of molecular coevolution in Protein–Protein interactions. Mol. Biol. Evol..

[17] Pazos F., Helmer-Citterich M., Ausiello G., Valencia A. (1997). Correlated mutations contain information about Protein–Protein interaction. J. Mol. Biol..

[18] Ofran Y., Rost B. (2007). ISIS: Interaction sites identified from sequence. Bioinformatics.

[19] Murakami Y., Mizuguchi K. (2010). Applying the Naïve Bayes classifier with kernel density estimation to the prediction of Protein–Protein interaction sites. Bioinformatics.

[20] Hamp T., Rost B. (2015). Evolutionary profiles improve Protein–Protein interaction prediction from sequence. Bioinformatics.

[21] Park Y., Marcotte E.M. (2012). Flaws in evaluation schemes for pair-input computational predictions. Nat. Methods.

[22] Hamp T., Rost B. (2015). More challenges for machine-learning protein interactions. Bioinformatics.

[23] Kawashima S., Kanehisa M. (2000). AAindex: Amino Acid index database. Nucleic Acids Res..

[24] Kawashima S., Pokarowski P., Pokarowska M., Kolinski A., Katayama T., Kanehisa M. (2008). AAindex: Amino acid index database, progress report 2008. Nucleic Acids Res..

[25] Nakai K., Kidera A., Kanehisa M. (1988). Cluster analysis of amino acid indices for prediction of protein structure and function. Protein Eng..

[26] Tomii K., Kanehisa M. (1996). Analysis of amino acid indices and mutation matrices for sequence comparison and structure prediction of proteins. Protein Eng..

[27] Gallet X., Charloteaux B., Thomas A., Brasseur R. (2000). A fast method to predict protein interaction sites from sequences1. J. Mol. Biol..

[28] Chou K.C. (2001). Prediction of protein cellular attributes using pseudo-amino acid composition. Proteins.

[29] Chou K.C., Cai Y.D. (2003). Predicting protein quaternary structure by pseudo amino acid composition. Proteins.

[30] Zhang S.W., Pan Q., Zhang H.C., Shao Z.C., Shi J.Y. (2006). Prediction of protein homo-oligomer types by pseudo amino acid composition: Approached with an improved feature extraction and Naive Bayes Feature Fusion. Amino Acids.

[31] Jia J., Liu Z., Xiao X., Liu B., Chou K.C. (2016). iPPBS-Opt: A sequence-based ensemble classifier for identifying Protein–Protein binding sites by optimizing imbalanced training datasets. Mol. (Basel, Switz.).

[32] Jia J., Liu Z., Xiao X., Liu B., Chou K.C. (2015). iPPI-Esml: An ensemble classifier for identifying the interactions of proteins by incorporating their physicochemical properties and wavelet transforms into PseAAC. J. Theor. Biol..

[33] Jia J., Liu Z., Xiao X., Liu B., Chou K.C. (2016). Identification of Protein–Protein binding sites by incorporating the physicochemical properties and stationary wavelet transforms into pseudo amino acid composition. J. Biomol. Struct. Dyn..

[34] Ashkenazy H., Erez E., Martz E., Pupko T., Ben-Tal N. (2010). ConSurf 2010: Calculating evolutionary conservation in sequence and structure of proteins and nucleic acids. Nucleic Acids Res..

[35] Celniker G., Nimrod G., Ashkenazy H., Glaser F., Martz E., Mayrose I. (2013). Consurf: Using evolutionary data to raise testable hypotheses about protein function. Israel J. Chem..

[36] Pupko T., Bell R.E., Mayrose I., Glaser F., Ben-Tal N. (2002). Rate4Site: An algorithmic tool for the identification of functional regions in proteins by surface mapping of evolutionary determinants within their homologues. Bioinformatics.

[37] Zhou H.X., Qin S. (2007). Interaction-site prediction for protein complexes: A critical assessment. Bioinformatics.

[38] Chou K.C. (2011). Some remarks on protein attribute prediction and pseudo amino acid composition. J. Theor. Biol..

[39] Chen W., Ding H., Feng P., Lin H., Chou K.C. (2016). iACP: A sequence-based tool for identifying anticancer peptides. Oncotarget.

[40] Chen W., Feng P., Ding H., Lin H., Chou K.C. (2016). Using deformation energy to analyze nucleosome positioning in genomes. Genomics.

[41] Jia J., Liu Z., Xiao X., Liu B., Chou K.C. (2016). iSuc-PseOpt: Identifying lysine succinylation sites in proteins by incorporating sequence-coupling effects into pseudo components and optimizing imbalanced training dataset. Anal. Biochem..

[42] Jia J., Liu Z., Xiao X., Liu B., Chou K.C. (2016). pSuc-Lys: Predict lysine succinylation sites in proteins with PseAAC and ensemble random forest approach. J. Theor. Biol..

[43] Jia J., Liu Z., Xiao X., Liu B., Chou K.C. (2016). iCar-PseCp: Identify carbonylation sites in proteins by Monte Carlo sampling and incorporating sequence coupled effects into general PseAAC. Oncotarget.

[44] Liu B., Fang L., Liu F., Wang X., Chou K.C. (2016). iMiRNA-PseDPC: MicroRNA precursor identification with a pseudo distance-pair composition approach. J. Biomol. Struct. Dyn..

[45] Liu B., Fang L., Long R., Lan X., Chou K.C. (2016). iEnhancer-2L: A two-layer predictor for identifying enhancers and their strength by pseudo *κ*-tuple nucleotide composition. Bioinformatics.

[46] Liu B., Long R., Chou K.C. (2016). iDHS-EL: Identifying DNase I hypersensitive sites by fusing three different modes of pseudo nucleotide composition into an ensemble learning framework. Bioinformatics.

[47] Liu Z., Xiao X., Yu D.J., Jia J., Qiu W.R., Chou K.C. (2016). pRNAm-PC: Predicting N^6^-methyladenosine sites in RNA sequences via physical-chemical properties. Anal. Biochem..

[48] Qiu W.R., Xiao X., Xu Z.C., Chou K.C. (2016). iPhos-PseEn: Identifying phosphorylation sites in proteins by fusing different pseudo components into an ensemble classifier. Oncotarget.

[49] Xiao X., Ye H.X., Liu Z., Jia J.H., Chou K.C. (2016). iROS-gPseKNC: Predicting replication origin sites in DNA by incorporating dinucleotide position-specific propensity into general pseudo nucleotide composition. Oncotarget.

[50] Ganganwar V. (2012). An overview of classification algorithms for imbalanced data sets. Int. J. Emerg. Technol. Adv. Eng..

[51] Gribskov M., Robinson N.L. (1996). Use of receiver operating characteristic (ROC) analysis to evaluate sequence matching. Comput. Chem..

[52] Liu R., Jiang W., Zhou Y. (2010). Identifying Protein–Protein interaction sites in transient complexes with temperature factor, sequence profile and accessible surface area. Amino Acids.

[53] Dhole K., Singh G., Pai P.P., Mondal S. (2014). Sequence-based prediction of Protein–Protein interaction sites with L1-logreg classifier. J. Theor. Biol..

[54] Chou K.C., Shen H.B. (2009). Recent advances in developing web-servers for predicting protein attributes. Nat. Sci..

[55] Chou K.C. (2015). Impacts of bioinformatics to medicinal chemistry. Med. Chem..

[56] Chen W., Lin H., Chou K.C. (2015). Pseudo nucleotide composition or PseKNC: An effective formulation for analyzing genomic sequences. Mol. BioSyst..

[57] Qiu W.R., Sun B.Q., Xiao X., Xu Z.C., Chou K.C. (2016). iHyd-PseCp: Identify hydroxyproline and hydroxylysine in proteins by incorporating sequence-coupled effects into general PseAAC. Oncotarget.

[58] Zhang C.J., Tang H., Li W.C., Lin H., Chen W., Chou K.C. (2016). iOri-Human: Identify human origin of replication by incorporating dinucleotide physicochemical properties into pseudo nucleotide composition. Oncotarget.

[59] Chen Y.K., Li K.B. (2013). Predicting membrane protein types by incorporating protein topology, domains, signal peptides, and physicochemical properties into the general form of Chou’s pseudo amino acid composition. J. Theor. Biol..

[60] Zhang J., Zhao X., Sun P., Ma Z. (2014). PSNO: Predicting cysteine S-nitrosylation sites by incorporating various sequence-derived features into the general form of Chou’s PseAAC. Int. J. Mol. Sci..

[61] Berman H.M., Westbrook J., Feng Z., Gilliland G., Bhat T.N., Weissig H., Shindyalov I.N., Bourne P.E. (2000). The protein data bank. Nucleic Acids Res..

[62] Chung J.L., Wang W., Bourne P.E. (2006). Exploiting sequence and structure homologs to identify Protein–Protein binding sites. Proteins.

[63] Yan C., Dobbs D., Honavar V. (2004). A two-stage classifier for identification of Protein–Protein interface residues. Bioinformatics.

[64] del Sol Mesa A., Pazos F., Valencia A. (2003). Automatic methods for predicting functionally important residues. J. Mol. Biol..

[65] Ezkurdia I., Bartoli L., Fariselli P., Casadio R., Valencia A., Tress M.L. (2009). Progress and challenges in predicting Protein–Protein interaction sites. Brief. Bioinform..

[66] Altschul S.F., Madden T.L., Schaffer A.A., Zhang J., Zhang Z., Miller W. (1997). Gapped blast and PSI-BLAST: A new generation of protein database search programs. Nucleic Acids Res..

[67] Dosztányi Z., Mészáros B., Simon I. (2009). ANCHOR: Web server for predicting protein binding regions in disordered proteins. Bioinformatics.

[68] Ansari S., Helms V. (2005). Statistical analysis of predominantly transient Protein–Protein interfaces. Proteins.

[69] Potenza E., Di Domenico T., Walsh I., Tosatto S.C.E. (2015). MobiDB 2.0: An improved database of intrinsically disordered and mobile proteins. Nucleic Acids Res..

[70] Schäffer A.A., Aravind L., Madden T.L., Shavirin S., Spouge J.L., Wolf Y.I., Koonin E.V., Altschul S.F. (2001). Improving the accuracy of PSI-BLAST protein database searches with composition-based statistics and other refinements. Nucleic Acids Res..

[71] Rao V.S., Srinivas K., Sujini G.N., Kumar G.N.S. (2014). Protein–Protein interaction detection: Methods and analysis. Int. J. Proteom..

[72] Mika S., Rost B. (2006). Protein–Protein interactions more conserved within species than across species. PLoS Comput. Biol..

[73] Bitbol A.F., Dwyer R.S., Colwell L.J., Wingreen N.S. (2016). Inferring interaction partners from protein sequences. Proc. Natl. Acad. Sci. USA.

[74] Li W., Godzik A. (2006). Cd-hit: A fast program for clustering and comparing large sets of protein or nucleotide sequences. Bioinformatics.

[75] Larkin M.A., Blackshields G., Brown N.P., Chenna R., McGettigan P.A., McWilliam H., Valentin F., Wallace I.M., Wilm A., Lopez R. (2007). Clustal W and Clustal X version 2.0. Bioinformatics.

[76] Felsenstein J. (2005). PHYLIP (Phylogeny Inference Package) Version 3.6.

[77] Janin J., Wodak S. (2007). The third CAPRI assessment meeting Toronto, Canada, April 20–21, 2007. Structure.

[78] Koike A., Takagi T. (2004). Prediction of protein–protein interaction sites using support vector machines. Protein Eng. Des. Sel..

[79] del Sol Mesa A., Pazos F., Valencia A. (2003). PhosphoSVM: Prediction of phosphorylation sites by integrating various protein sequence attributes with a support vector machine. J. Mol. Biol..

[80] Johansson F., Toh H. (2010). A comparative study of conservation and variation scores. BMC Bioinform..

[81] Karlin S., Brocchieri L. (1996). Evolutionary conservation of *RecA* genes in relation to protein structure and function. J. Bacteriol..

[82] Capra J.A., Singh M. (2007). Predicting functionally important residues from sequence conservation. Bioinformatics.

[83] Sander C., Schneider R. (1991). Database of homology-derived protein structures and the structural meaning of sequence alignment. Proteins.

[84] Kim H., Park H. (2003). Protein secondary structure prediction based on an improved support vector machines approach. Protein Eng..

[85] Wang Z., Zhao F., Peng J., Xu J. (2011). Protein 8-class secondary structure prediction using conditional neural fields. Proteomics.

[86] Petersen B., Petersen T.N., Andersen P., Nielsen M., Lundegaard C. (2009). A generic method for assignment of reliability scores applied to solvent accessibility predictions. BMC Struct. Biol..

[87] Atchley W.R., Zhao J., Fernandes A.D., Drüke T. (2005). Solving the protein sequence metric problem. Proc. Natl. Acad. Sci. USA.

[88] Hansen J.C., Lu X., Ross E.D., Woody R.W. (2006). Intrinsic protein disorder, amino acid composition, and histone terminal domains. J. Biol. Chem..

[89] Forman-Kay J.D., Mittag T. (2013). From sequence and forces to structure, function, and evolution of intrinsically disordered proteins. Struct. (Lond. Engl. 1993).

[90] Momen-Roknabadi A., Sadeghi M., Pezeshk H., Marashi S.A. (2008). Impact of residue accessible surface area on the prediction of protein secondary structures. BMC Bioinform..

[91] Bordner A.J., Abagyan R. (2005). Statistical analysis and prediction of Protein–Protein interfaces. Proteins.

[92] Sikić M., Tomić S., Vlahovicek K. (2009). Prediction of Protein–Protein interaction sites in sequences and 3D structures by random forests. PLoS Comput. Biol..

[93] Vapnik V.N. (1995). The Nature of Statistical Learning Theory.

[94] You Z.H., Lei Y.K., Zhu L., Xia J., Wang B. (2013). Prediction of Protein–Protein interactions from amino acid sequences with ensemble extreme learning machines and principal component analysis. BMC Bioinform..

[95] Das R., Dimitrova N., Xuan Z., Rollins R.A., Haghighi F., Edwards J.R., Ju J., Bestor T.H., Zhang M.Q. (2006). Computational prediction of methylation status in human genomic sequences. Proc. Natl. Acad. Sci. USA.

[96] Cai Y.D., Liu X.J., Xu X.B., Chou K.C. (2002). Support vector machines for prediction of protein subcellular location by incorporating quasi-sequence-order effect. J. Cell. Biochem..

[97] Chang C.C., Lin C.J. (2011). LIBSVM: A library for support vector machines. ACM Trans. Intell. Syst. Technol..

